# Combined use of maxillomandibular swing approach and neurosurgical ultrasonic aspirator in the management of extensive clival chordoma: A case report

**DOI:** 10.1186/1752-1947-2-49

**Published:** 2008-02-18

**Authors:** Shahid Hassan, Jafri M Abdullah, Shah Jihan Wan Din, Zamzuri Idris

**Affiliations:** 1 Department of ORL-HNS, School of Medical Sciences, Health Campus, Universiti Sains Malaysia, 16150 Kota Bharu, Kelantan, Malaysia; 2 Department of Neuroscience, School of Medical Sciences, Health Campus, Universiti Sains Malaysia, 16150 Kota Bharu, Kelantan, Malaysia

## Abstract

**Introduction:**

Chordoma is a rare malignant tumour with an incidence of metastasis of less than 10 percent. Usually arising from clivus its posterior extension may involve the brainstem before presenting as nasal mass and obstruction. Surgery is the main mode of treatment with adjuvant radiotherapy. However surgery is rarely possible for a large intracranial lesion.

**Case presentation:**

We report the case of an adolescent patient with a chordoma extending posteriorly to the brainstem and anteriorly to the nasopharynx and managed by the combination of resection using a maxillomandibular swing approach and the use of a neurosurgical ultrasonic aspirator.

**Conclusion:**

Maxillomandibular swing approach provides good access for large nasopharyngeal tumour extending brainstem area.

## Introduction

Chordomas are comparatively slow growing malignant neoplasms derived from notochord. They can present anywhere from skull base to sacrum. In the cranial region the tumours usually arises from the clivus. Clival chordomas usually present in the third and fourth decades of life and there is slight male preponderance [1]. Chordomas in children and adolescents are rarer and carry a worse prognosis. Hoch et al. (2006) in their series of skull base chordomas in adolescents found an overall survival rate of 81% [2].

Skull base chordomas usually extend posteriorly from the clivus to the sella turcica and brain stem [3]. Although it is a locally aggressive tumour, distant metastasis is rare. From the spheno-occipital region it often protrudes into the nasopharynx. Due to the large hidden space in the nasopharynx, clinical presentation is usually late and associated with central nervous system deficit. This tumour can be confused with chondrosarcoma but it is characterized by positive immunohistochemistry to vimentin, S-100 protein, and epithelial markers, namely keratin and EMA (Epithelial Membrane Antigen) [4]. Treatment usually consists of aggressive surgical resection but external beam radiotherapy, proton beam therapy or gamma knife radiosurgery are alternatives that can be used in tumours with extensive intracranial extension [5]. Complete surgical resection with or without proton beam radiotherapy remains the treatment of choice [6]. However even with proton beam radiotherapy, chordomas respond less favourably when compare to chondrosarcomas [5]. Here we present the case of an adolescent patient with a large clival chordoma with resection using a maxillomandibular swing approach and ultrasonic aspirator.

## Case presentation

A 17 year old boy presented with progressively worsening right-sided nasal blockage of one year duration, with two episodes of epistaxis and deterioration of vision. On examination there was a mass in the right nasal cavity extending across the postnasal space on either side. Fundoscopy revealed signs of left eye compression and optic neuropathy. An initial differential diagnosis was of chordoma, angiofibroma, pyogenic granuloma and nasopharyngeal carcinoma.

**Figure 1:**
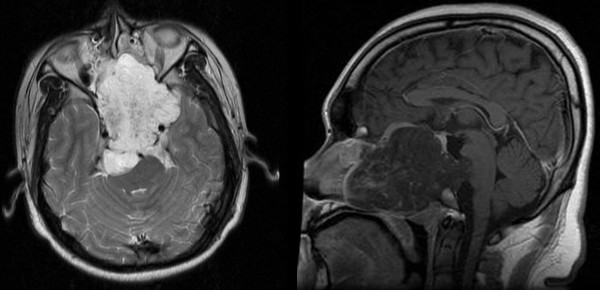
**Sagittal and axial views of brain MRI scan**. Image shows tumour in the nasopharynx extending from nasal cavity to brainstem posteriorly.

**Figure 2:**
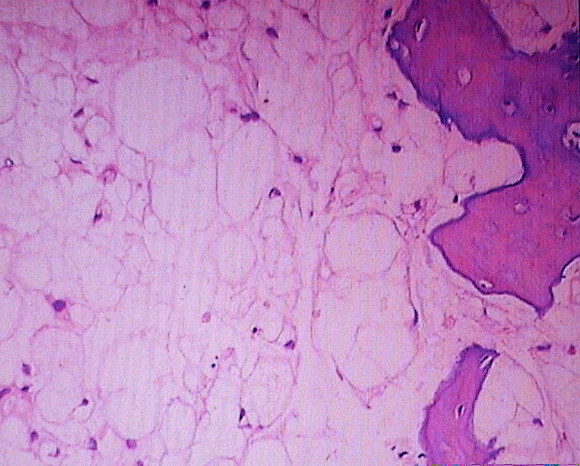
**Histopathology slide**. Typical physaliferous cells seen with bone destruction.

CT (Computed Tomography) scan showed a hypodense mass in the nasopharynx entering into the sellar and parasellar regions with erosion of the pituitary fossa, clivus and middle cranial fossa. An MRI (Magnetic Resonance Imaging) brain sequence showed a large clival based tumour with involvement of the dura at the region of the whole clivus where the tumour had compressed the brain stem anteriorly (Fig. 1). Cerebral angiogram revealed a nonvascular mass with blood supply coming from both maxillary arteries. A definite diagnosis of chordoma was made on histopathological examination of a biopsy taken from the nasopharyngeal mass which showed lobules of typical physaliferous cells (positive for cytokeratin, vimentin and S100) destroying the bone trabeculae (Fig. 2).

Tumour excision was via left maxillary swing, left mandibular swing and midline tongue split followed by reassembly and filling of the defect utilizing rectus abdominus muscle. First a left maxillary swing approach via a Weber-Fergusson-Longmire incision was performed. The maxilla was swung laterally based on a cheek flap. As the access to the tumour, which was extending from skull base to first cervical vertebra, was not adequate with this approach alone, a paramedian mandibulotomy facilitating a left mandibullar swing was also performed. However a massive tongue occupying the mid part of the dissection did not allow free instrumentation. A temporary midline glossotomy was carried out to provide more exposure. This ultimately gave wide access to the whole clival mass.

The chordoma was resected using an ultrasonic aspirator set at a high frequency mode of 100 Hertz which is typically used in meningioma resections. The tip of the Dissectron ultrasonic aspirator (Satelec Medical, Bordeaux, France) was calibrated with the Omnisight Image Guided System (Radionics, Burlington, Massachusetts) so that precise removal could be performed without injury of the structures within the dural space i.e. brainstem, basilar artery and pituitary gland. Despite this there was a small dural tear at the level of the upper clivus where invasion was maximum. At the end of the surgical procedure, the defect was filled with a free rectus abdominis muscle transfer. Despite this a CSF (Cerebrospinal Fluid) fistula developed and was managed by external ventricular drainage which was converted to a low pressure ventricular peritoneal shunt fourteen days later. The CSF leak stopped two days two days after surgery and acetazolamide was continued for duration of 5 days.

## Discussion

Chordomas are lobulated and apparently capsulated tumours which arise from notochord and derive from ectoderm. Mainly seen in the sacrococygeal region, they may arise from the spheno-occipital region and protrude into the nasopharynx. While plain X-rays may show tumour with destruction of the clivus, CT-scan and MRI are essential assessment tools in delineating the gross margins of a chordoma [7]. Morphologically they can be confused with chondrosarcomas but they are characterised by bubble cells (physaliferous cells) with strands of spindle-shaped cells [8]. Immunohistochemistry is of diagnostic value and the tumour is stained positive to S-100, vimentin, epithelial membrane antigen and cytokeratin antibodies [5].

While radical surgical resection is the treatment of choice, this is rarely possible due to intracranial extension. There have been reports of using an endoscopic approach but in cases with extensive dural invasion, inferior clivus-centred tumours and large tumours extended to the occipital condyle, an open external approach is preferred [6]. Radiotherapy has a dose-effect relationship and meticulous technique is required to achieve high dosage safety in tumours abutting the central nervous system [9].

Our patient was an adolescent boy with extensive chordoma and we faced similar problems if we were to approach the tumour transcranially. Furthermore the proximity of vital structures such as internal carotid arteries and cranial nerves made this surgical approach difficult and very challenging. However we succeeded in approaching the tumour via a maxillomandibular swing with a tongue split procedure. This procedure provided adequate exposure for surgical resection of the tumour mass utilizing an ultrasonic aspirator to remove tumour which was abutting brainstem without any residual central nervous system deficit. The advantage of the use of an ultrasonic aspirator is its ability to remove tumour without causing damage to nearby blood vessels. Radiotherapy was subsequently administered and the patient is regularly followed-up and has been in sound health for three years from the time of surgery.

## Conclusion

Excision of an extensive chordoma with minimal complications and a good prognosis is possible utilizing an upper & lower jaw double swing approach in association with neurosurgical expertise.

## Abbreviations

CSF – cerebrospinal fluid

EMA – epithelial membrane antigen

CT – Computed Tomography

MRI – Magnetic Resonance Imaging

## Competing interests

The author(s) declare that they have no competing interests.

## Authors' contributions

SH: Drafted the overall design of the manuscript, the otolaryngology management part and collect all the figures and photos. JMA: Drafted the neurosurgical part of the management. SJWD: Drafted the clinical presentation and acquired references. ZI: Help in drafting the overall manuscript and performed final review. All authors have read and approved the final manuscript.

## Consent

Written informed consent was obtained from the patient for publication of this case report and any accompanying images. A copy of the written consent is available for review by the Editor-in-Chief of this journal.

## References

[B1] Matsumoto J Towbin RB Ball WS Jr. Cranial chordomas in infancy and childhood. A report of two cases and review of the literature Pediatr Radiol 1989 20 1-2 28 32 10.1007/BF02010629 2689982

[B2] Hoch BL Nielsen GP Liebsch NJ Rosenberg AE Base of skull chordomas in children and adolescents: a clinicopathologic study of 73 cases Amer J Surg Pathology 2006 30 7 811 818 10.1097/01.pas.0000209828.39477.ab 16819322

[B3] Utne JR Pugh DG The roentgenologic aspects of chordoma Am J Roentgenol Radium Ther Nucl Med 1955 74 4 593 608 13258915

[B4] Rosenberg AE Nielsen GP Keel SB Renard LG Fitzek MM Munzenrider JE Liebsch NJ Chondrosarcoma of the base of the skull: a clinicopathologic study of 200 cases with emphasis on its distinction from chordoma Am J Surg Pathol 1999 23 11 1370 1378 10.1097/00000478-199911000-00007 10555005

[B5] Tzortzidis F Elahi F Wright D Natarajan SK Sekhar LN Patient outcome at long-term follow-up after aggressive microsurgical resection of cranial base chordomas Neurosurgery 2006 59 2 230 7; discussion 230-7 10.1227/01.NEU.0000223441.51012.9D 16883163

[B6] Frank G Sciarretta V Calbucci F Farneti G Mazzatenta D Pasquini E The endoscopic transnasal transsphenoidal approach for the treatment of cranial base chordomas and chondrosarcomas Neurosurgery 2006 59 1 Suppl 1 ONS50 7; discussion ONS50-7 16888551 10.1227/01.NEU.0000219914.17221.55

[B7] Oot RF Melville GE New PF Austin-Seymour M Munzenrider J Pile-Spellman J Spagnoli M Shoukimas GM Momose KJ Carroll R The role of MR and CT in evaluating clival chordomas and chondrosarcomas Am J Roentgenol 1988 151 3 567 575 3261519 10.2214/ajr.151.3.567

[B8] Hellquist HB Pathology of the Nose and Paranasal Sinus 1990 London , Butterworth-Heinemann Medical

[B9] Castro JR Collier JM Petti PL Nowakowski V Chen GT Lyman JT Linstadt D Gauger G Gutin P Decker M Charged particle radiotherapy for lesions encircling the brain stem or spinal cord International Journal of Radiation Oncology, Biology, Physics 1989 17 3 477 484 2506156 10.1016/0360-3016(89)90097-7

